# Stepfamily Dynamics and Emerging Adults' Adjustment in Japan: Four Patterns Affecting Stepchildren's Outcomes

**DOI:** 10.1111/famp.70071

**Published:** 2025-09-26

**Authors:** Yasumitsu Jikihara, Mari Kikuchi, Shinji Nozawa

**Affiliations:** ^1^ Graduate School of Human Sciences The University of Osaka Osaka Japan; ^2^ Faculty of Economics Osaka Sangyo University Osaka Japan; ^3^ Department of Sociology Meiji Gakuin University Tokyo Japan

**Keywords:** emerging adults, latent profile analyses, parenting, stepfamily, stepparenting

## Abstract

Stepfamilies are increasingly prevalent worldwide; however, research on non‐Western stepfamilies remains limited. This study examines stepparenting, parenting behaviors, stepcouple relationships, and biological parents' co‐parenting quality in Japan, exploring their impact on the psychological adjustment of emerging adults (EAs). The sample included 421 Japanese EAs (aged 20–29) raised in stepfamilies with a resident biological mother and stepfather and a nonresident biological father. Using latent profile analysis, we identified four relationship patterns: Residence‐Centered (37.8%) with strong resident mother**–**stepfather ties, Inclusive (15.9%) with positive bonds across all parental figures, Inter‐household Ambivalent Loyalty (22.8%) with an ambivalent nonresident father bond and biological parents' conflictive co‐parenting, and High Stepfamily Conflict (23.5%) with frequent residential stepfamily conflict. The latter two profiles were associated with low self‐esteem, high depression and anxiety, and increased aggression. Similar patterns in Western studies suggest that the key aspects of stepfamily functioning may be shared across cultural contexts. Our findings suggest that balanced parental involvement and positive stepparent relationships may influence stepfamily adaptation. Providing effective support for stepfamilies requires assessing the entire family system—including nonresidential biological parents—rather than focusing solely on individual relationships. Positive parenting and connections are important; however, we found that differences in adjustment were more strongly linked to negative relational features. Hence, interventions that reduce these negative dynamics may benefit families facing considerable difficulties. Clinicians should attempt to deepen their understanding of what does and does not work in stepfamily settings.

## 1 Introduction

Stepfamilies are becoming more common in the United States and globally (Australian Bureau of Statistics 2021; Payne 2019). Although official statistics are unavailable in Japan, the increasing remarriage rate suggests a corresponding rise in stepfamilies (Ministry of Health, Labour and Welfare in Japan 2024). Research on stepfamilies has primarily focused on the impact of stepfamily dynamics on children's development, with increasing recognition that these effects continue into emerging adulthood (Coleman et al. 2000; Egginton et al. 2021). Early stepfamily research often relied on a deficit comparison model, emphasizing negative differences between stepfamilies and non‐stepfamilies (Coleman et al. 2000). This approach has since been critiqued for overlooking the diversity and complexity of stepfamily life. Recent studies have shifted toward a resilience‐focused perspective, emphasizing within‐family processes that foster either positive or negative adjustment in stepchildren (Ganong and Coleman 2018; Ganong et al. 2025). This shift recognizes that children's experiences in stepfamilies vary widely, highlighting the importance of identifying family dynamics and contextual factors that promote resilience in order to develop effective support and intervention strategies (Ganong and Coleman 2017; Saint‐Jacques et al. 2018).

Researchers have emphasized the influence of parent–child and stepparent‐stepchild relationships on stepchildren's adjustment and the importance of understanding these relationships from an interactional perspective rather than examining individual effects in isolation (Coleman et al. 2000; Ganong and Sanner 2023). Recent studies have identified specific stepparenting and parenting practices that effectively influence parent–child as well as stepparent‐stepchild relationships (Ganong et al. 2025; Ganong, Coleman, et al. 2021; Sanner et al. 2022). However, stepfamily dynamics extend beyond the parent–child dyad, encompassing stepcouple relationships and biological parents' co‐parenting arrangements, all of which impact a stepchild's adjustment (Ganong et al. 2025; Ganong, Sanner, et al. 2021; Jensen 2017).

Despite several empirical studies on Western stepfamilies, studies on non‐Western stepfamilies, including Japan, remain limited (Ganong and Sanner 2023). This study aims to identify and categorize effective stepparenting and parenting behaviors, stepcouple relationships, and biological parents' co‐parenting practices in Japan, examining their associations with psychological adjustment among emerging adults (EAs).

### 1.1 Patterns of Parent–Child Relationships

Common challenges in stepfamilies include family‐boundary ambiguity, co‐parent conflicts, stepparent‐stepchild tensions, differing parenting styles, conflicting family cultures, frequent relocations, and declines in the quality of parent–child dyadic relationships (Ganong and Coleman 2017; Papernow 2013). To address these challenges, cultivating high‐quality dyadic relationships is essential, as they promote youth adjustment and enhance family cohesion (Cartwright and Seymour 2002; Ganong and Coleman 2017).

However, findings from studies examining the quality of dyadic relationships are inconsistent. In particular, the influence of the nonresidential biological parent relationship shows significant variation (King 2007; Jensen et al. 2017). The absence of a comprehensive perspective on all three parental figures may account for the considerable variability in research on (step) parent–child relationships in stepfamilies. In recent years, studies that categorize stepchildren's relationships with all three key adults—the two biological parents and the stepparent—have become more common (Amato et al. 2016; Egginton et al. 2021; Jensen 2017; King 2006; Zhao et al. 2024). According to Ganong and Sanner (2023), these studies identified four dyadic relationship types. Stepchildren in the Close to Both or All category, who maintain close relationships with biological parents and the stepparent, showed the most positive outcomes. Those in the Close to Neither or None category, who lack close relationships with any parental figures, experienced the poorest outcomes. Stepchildren in the Substitution category, who have a strong bond with their residential stepparent but not with their nonresidential biological parent, exhibited positive adjustment. The Retention category, characterized by closeness to the nonresidential biological parent but not the stepparent, was less common and associated with moderate outcomes. Overall, close relationships with all parental figures were associated with the most positive outcomes, whereas the absence of such relationships was linked to poor adjustment.

### 1.2 Effective Stepparenting and Parenting Behaviors

Previous studies have assessed the quality of relationships in stepfamilies using key concepts such as closeness and warmth and have often classified these relationships into typologies based on these factors. However, researchers highlight the importance of effective stepparenting and parenting in shaping relationship quality (Ganong et al. 2025; Ganong, Coleman, et al. 2021; Sanner et al. 2022). Effective stepparenting behaviors involve two key aspects. First, developing positive stepparent–stepchild bonds includes affinity‐seeking behaviors, caregiving, communicating effectively, using indirect relationship‐bonding strategies, and stepchildren's receptivity to the stepparent's bonding efforts. Second, establishing clear stepparent roles is essential for navigating family dynamics (Ganong, Coleman, et al. 2021). Effective parenting behaviors include maintaining strong parent–child bonds, setting appropriate parent–child communication boundaries, exercising parental control, supporting stepparent–stepchild relationship development, and facilitating stepfamily cohesion (Sanner et al. 2022). In summary, relationship quality in stepfamilies is strongly influenced by effective stepparenting and parenting behaviors. However, how these combined behaviors relate to stepchildren's outcomes remains poorly understood.

### 1.3 Stepcouple and Co‐Parenting Relationships

In stepfamilies, both stepcouple and parent–child relationships are crucial. Unlike couples in biological families, stepcouples must navigate their relationship while adapting to an existing family structure, making integration especially challenging (Papernow 2013). Jensen (2017) found that children in stepfamilies with dissatisfied and conflictual stepcouple relationships (the Unhappy Couple pattern) are more likely to engage in delinquent behavior than those in families where stepcouples have higher relationship quality and greater involvement of the nonresident parent (the Inclusive pattern).

Additionally, stepfamily dynamics depend heavily on the interactions between residential and nonresidential biological parents. The formation of stepfamilies brings shifting roles and rules, making effective co‐parenting a particularly complex challenge (Ganong et al. 2025; Ganong, Sanner, et al. 2021). Moreover, co‐parenting conflict following divorce is one of the strongest predictors of children's maladjustment (Amato 2010). However, despite their relevance to stepchildren's outcomes, these relationships have received relatively little attention in previous research (Ganong, Coleman, et al. 2021; Ganong, Sanner, et al. 2021; Jensen 2017).

### 1.4 Theoretical Framework

Building upon prior research, multiple theoretical frameworks contribute to a nuanced and inclusive comprehension of dyadic relationships in stepfamilies. Family systems theory suggests that individual behaviors and outcomes are influenced by interrelated family dynamics, indicating that alterations in one component of the system can impact the entire familial structure (Cox and Paley 2003). Consequently, a thorough understanding of stepfamily dynamics can be deepened through the concurrent analysis of multiple individuals and their interrelationships. To clarify the dynamics of stepfamilies, we use three types of behaviors—stepparenting, residential, and nonresidential parenting—and two key parental relationships—the quality of stepcouple relationships and biological parents' co‐parenting relationships.

The cultural and institutional context in Japan introduces unique dimensions to stepfamily dynamics. Ironically, in the historical process of Japan's modernization or Westernization, divorce and remarriage have been stigmatized (Nozawa 2008, 2020), leading to the social marginalization of stepfamilies as an “invisible” family form (Nozawa 2008). This invisibility is further reinforced by the lack of a comprehensive term for stepfamilies in the Japanese language. Additionally, Japan's legal framework exacerbates these challenges through its lack of parenting plans or parent education programs, sole‐custody systems, and permissive stepparent adoption practices. For example, 87.7% of divorces are based only on mutual agreement (Ministry of Health, Labour and Welfare in Japan 2024), only one parent can be granted legal custody of the child after divorce, and the custodial parent's new spouse can adopt the child without the noncustodial parent's consent. These practices often result in the loss of contact between noncustodial parents and their children, often leading to strained family relationships. Evidence‐based research on custodial mothers following divorce, revealed that merely 32.7% of noncustodial fathers maintain contact with their children (Ministry of Health, Labour and Welfare in Japan 2022). In this institutional context, Nozawa (2015a, 2020) theorized the traditionally normative pattern as the “scrap and build” household model. This model conceptualizes stepfamily formation as dismantling the previous family structure and constructing a new nuclear family unit consisting of the custodial parent, the child, and the stepparent. It also describes closed household boundaries, where family is narrowly defined and the noncustodial parent is typically replaced by the stepparent. These cultural expectations and institutional factors create unique stressors for stepfamilies, resulting in social pressure and internal adaptation, ultimately shaping their complex family dynamics.

### 1.5 The Present Study

The first objective of this study is to analyze the patterns of stepparenting, parenting behaviors, and quality of stepcouple and biological parents' co‐parenting relationships. In Japan, approximately 85% of mothers receive sole custody after divorce. Thus, this study focuses on EAs raised in stepfamilies where the biological mother and stepfather cohabit, while the biological father resides separately. This deliberate focus ensures a sufficient sample size and allows for a more detailed examination of the most common stepfamily structure in Japan. To further assess effective and ineffective stepparenting and parenting behaviors, we developed new scales and assessed their association with the Parental Bonding Instrument (Parker et al. 1979). These patterns and various sociodemographic factors (e.g., EAs' gender, age at parental divorce, and the presence of siblings) were also analyzed. Finally, the relationship between these patterns and EAs' psychological outcomes (self‐esteem, depression/anxiety, and aggression) was analyzed.

## 2 Method

### 2.1 Participants and Procedure

This cross‐sectional and retrospective survey of 444 Japanese EAs (aged 20–29) was conducted using a community‐based sample of Japanese adults who had registered as potential respondents with an Internet research company (Macromill Inc.) in June 2022. Participants met the following inclusion criteria: (a) having divorced biological parents and residing with the biological mother, (b) having a remarried or cohabited biological mother and living with a stepfather before the age of 15, and (c) all parental figures (biological mother, biological father, and stepfather) were alive. Participants were informed that their participation was voluntary. The Ethics Committee of the University of Toyama approved this study (number 010).

Data from 23 participants were excluded because of unnatural or inconsistent response patterns (e.g., repetitive answers such as “1, 1, 1…”). Of the remaining 421 participants, 105 (24.9%) were male and 315 (74.8%) were female. Their ages ranged from 20 – 29 years, with a mean age of 24.6 years (SD = 2.70). Approximately one‐third (29.7%) of the participants had enrolled in a bachelor's degree program. A substantial proportion of participants (43.7%) were married. Age at entry into the stepfamily ranged from 0 – 15 years, with a mean age of 8.92 years (SD = 3.65). The year of stepfamily entry ranged from 1993 – 2017, with a mean year of 2006 (SD = 4.60).

### 2.2 Measures

#### 2.2.1 Stepparenting and Parenting Behaviors

First, the first author reviewed the literature on effective and ineffective stepparenting and parenting behaviors in English or Japanese published up to 2020 and extracted specific behaviors. Similar behaviors were then grouped into categories to develop a categorical framework. Next, the co‐authors provided feedback and made revisions. The final categories, after review, are presented in Table S1. Effective stepparenting was divided into two categories: Engaging in Affinity‐Seeking (Ganong et al. 1999; Stoll et al. 2006; e.g., “My stepfather talked to me one‐on‐one.”) and Advocating for the Stepchild (Kinniburgh‐White et al. 2010; e.g., “My stepfather intervened when my mother and I disagreed.”). The first category of effective parenting, common to both residential and nonresidential parents, was Maintaining Close Parent–Child Bonds (Cartwright and Seymour 2002; Nozawa 2015b; Stoll et al. 2006). Its examples include Displaying Warmth (e.g., “My father tried to understand me.”) and Protecting One‐on‐One Time (e.g., “I felt that my mother prioritized spending time with me.”). The second category, unique to residential parents, was Supporting Stepparent–Child Relationship Development (Cartwright and Seymour 2002; Nozawa 2015b; Stoll et al. 2006; e.g., “My mother intervened when my stepfather and I disagreed.”). Conversely, ineffective stepparenting and parenting involved Replacing the Nonresidential Biological Parent with the Stepparent (Nozawa 2015b; e.g., “My mother asks me to call my stepfather ‘Dad’.”), and Imposing Rules and Values (Kinniburgh‐White et al. 2010; Nozawa and Kikuchi 2014; e.g., “My stepfather made rules and attempted to force them on me.”). The latter has been identified as a characteristic of ineffective stepparenting, particularly when stepparents assume disciplinary authority early in the relationship (Ganong et al. 2025). While such behaviors reflect authoritarian parenting—a style associated with poor outcomes in both biological and stepparent contexts (Masud et al. 2019)—they may be especially detrimental in stepfamily settings. Therefore, although we applied this item to all parental figures, its interpretation should take these contextual risks into account.

Items appropriate to the above categories were created separately for stepfathers, residential mothers, and nonresidential fathers, and were discussed and revised by the coauthors. The final number of items was 19 for stepfathers, 20 for residential mothers, and 10 for nonresidential fathers. Participants were asked to recall the period from when their stepfather and biological mother started living together until they were 18 years old and to respond using a 4‐point scale (1 = not at all, 2 = not very much, 3 = some, 4 = quite a lot). Additionally, as participants might have found it difficult to answer questions about the biological father if there was no interaction at all, we included the option “I don't know because there was no interaction at all.” The response with the lowest value was “1: not at all,” following Amato et al. (2016).

#### 2.2.2 Parental Bonding

The Parental Bonding Instrument (PBI; Parker et al. 1979) was validated by Kitamura and Suzuki (1993) for use with Japanese populations, with most findings showing the three factors of Care, Denial of Psychological Autonomy, and Encouragement of Behavioral Freedom, instead of the original two factors of Care and Protection (Narita et al. 2000). In this study, a three‐factor structure was assumed, and 10 items were selected from each factor. Participants provided their responses using a four‐point scale ranging from 1 (disagree) to 4 (agree).

#### 2.2.3 Frequency of Father–Child Visitation

Participants were asked to report how frequently they had visited their biological father following their parents' divorce. For each of the four age periods—(a) before entering elementary school, (b) elementary school, (c) junior high school, and (d) high school—they selected one of the following options: (0) never, (1) about once during this period, (2) once a year, (3) once every few months, (4) once a month, (5) twice a month, or (6) once a week or more. The average score across all reporting periods was used as the visitation frequency score for each period.

#### 2.2.4 Stepcouple Relationship Quality

A strong stepcouple relationship was measured using the Quality Marriage Index (QMI; Norton 1983), which was translated into Japanese and has been used with Japanese populations (Moroi 1997). Four items were selected in this study. A conflictive stepcouple relationship was measured using the Adolescents' Perception of Marital Conflict Scale in Japanese (Yamamoto and Ito 2012). Four items were selected from each conflict intensity subscale, and our original item was added (“My mother and stepfather disagreed about my lifestyle and habits.”). Responses were given using a four‐point scale ranging from 1 (disagree) to 4 (agree). Cronbach's *α* coefficients were 0.82 for a strong and 0.89 for a conflictive stepcouple relationship.

#### 2.2.5 Biological Parents' Co‐Parenting Quality

Biological parents' co‐parenting was assessed using the Children's Perception of Co‐parenting following separation or divorce (Jikihara and Ando 2019). We selected two items from the Parent's Trust and Support subscale (Cooperative Co‐parenting) and eight items from Parental Denigration, Caught between Parents, and Inter‐Parental Conflict subscales (Conflictive Co‐parenting). Participants responded on a five‐point scale ranging from 1 (disagree) to 4 (agree). Cronbach's *α* coefficients were 0.80 for cooperative co‐parenting and 0.90 for conflictive co‐parenting.

#### 2.2.6 Self‐Esteem

Rosenberg's Self‐Esteem Scale (RSES; Rosenberg 1979) was validated for Japanese populations by Yamamoto et al. (1982). This scale assesses relatively stable perceptions of overall self‐worth. Participants responded on a five‐point scale, with options ranging from 1 (disagree) to 5 (agree). Cronbach's *α* coefficient was 0.82.

#### 2.2.7 Psychological Distress

The Kessler Psychological Distress Scale (K10; Kessler et al. 2003) was validated for Japanese populations by Furukawa et al. (2003). This scale is extensively utilized for evaluating symptoms associated with depression and anxiety, effectively distinguishing between individuals with serious mental illness and those without. Participants provided their responses using a five‐point scale, with options ranging from 0 (none of the time) to 4 (all of the time). Cronbach's *α* coefficient was 0.96.

#### 2.2.8 Proactive and Reactive Aggression

The Japanese version of the Proactive and Reactive Aggressiveness Scale for University Students (SPRAS‐U) was developed and validated by Hamaguchi (2017). This scale assesses two types of aggression: proactive aggression, which is calculated, emotionally detached (“cold‐blooded”), and goal‐oriented, occurring without provocation (Romero‐Martínez et al. 2022); and reactive aggression, which is impulsive and driven by negative emotions (“hot‐blooded”) in response to provocation. Five items were selected from each aggression type. Items assessing proactive aggression were drawn from the Irritability and Retaliatory Intent subscales, while those measuring reactive aggression were drawn from the Competence to Attack and Adherence to Desire subscales. Responses were given using a five‐point scale ranging from 1 (disagree) to 5 (agree). Cronbach's *α* coefficients were 0.69 for proactive aggression and 0.85 for reactive aggression.

### 2.3 Analytic Plan

Stepparenting and parenting behaviors were examined using exploratory factor analysis (EFA) with maximum likelihood estimation and Promax rotation. The number of factors was determined using eigenvalues, explained variance, and scree tests. Items with low factor loading (< 0.4) or high cross‐loadings (> 0.3) were removed, and EFA was repeated until all remaining items met the criteria. Cronbach's *α* coefficients were then calculated to assess internal consistency. Partial correlations were conducted to examine the validity of the factors of the well‐established Parental Bonding Instrument. These analyses were performed using IBM SPSS Version 28.

Latent profile analysis (LPA) was conducted in Mplus 8.4 to identify distinct participant profiles based on stepparenting and parenting behaviors, frequency of father–child visitation, stepcouple relationship quality, and biological parent's co‐parenting quality. The best‐fitting model was determined using Akaike information criterion (AIC), Bayesian information criterion (BIC), adjusted BIC (aBIC), and likelihood ratio tests (Vuong‐Lo‐Mendell‐Rubin likelihood ratio test [VLRT], Lo‐Mendell‐Rubin likelihood ratio test [LMR], and Bootstrap Likelihood Ratio Test [BLRT]; Nylund et al. 2007). The optimal solution was selected based on fit indices, interpretability, and profile size (≥ 5% of the sample), and entropy values (> 0.80) were used to evaluate classification accuracy. Predictors of class membership were examined using the R3STEP method (Asparouhov and Muthén 2014), and odds ratios (OR) with 95% confidence intervals (CIs) were reported. Results were considered statistically significant when the 95% CI did not include 1. The BCH method was used to analyze distal outcomes (self‐esteem, depression and anxiety, and aggression) via Wald chi‐square tests while accounting for mean and variance differences across profiles (Asparouhov and Muthén 2021).

## 3 Results

### 3.1 Development of Stepparenting and Parenting Behaviors Scale

#### 3.1.1 Stepfathers' Stepparenting Behaviors

EFA was conducted using an initial set of 19 items. Problematic items were then removed sequentially until a three‐factor model, consisting of 17 items, emerged. The loadings of the items are shown in Table S2. Factor 1, Engaging in Affinity‐Seeking and Advocating for the Stepchild, consisted of eight items with factor loadings ranging from 0.71 – 0.86 (*α* = 0.93). These behaviors involved engaging in one‐on‐one interactions with the stepchild and mediating between the resident biological mother and the stepchild. Factor 2, Replacing the Nonresidential Biological Father, consisted of five items with factor loadings ranging from 0.60 – 0.79 (*α* = 0.85). These indicators included expressions of negative attitudes toward the topic of the biological father and restricting parenting time with the biological father. Factor 3, Imposing Rules and Values, consisted of four items with factor loadings ranging from 0.58 – 0.85 (*α* = 0.87). These indicators included imposing values and rules and being strict with children.

#### 3.1.2 Residential Mothers' Parenting Behaviors

EFA was conducted using an initial set of 20 items. Items with low factor loadings, high cross‐loadings, and/or low communalities were marked as candidates for removal. Problematic items were removed sequentially until a three‐factor model, consisting of 18 items, emerged. The item loadings are shown in Table S3. Factor 1, Maintaining Close Parent–Child Bonds and Supporting Stepparent–Child Relationships, consisted of nine items with factor loadings ranging from 0.70 – 0.83 (*α* = 0.92). These behaviors included protecting one‐on‐one time, displaying warmth, and supporting stepparent–child relationship development. Factor 2, Replacing the Nonresidential Biological Father, consisted of five items with factor loadings ranging from 0.41 – 0.63 (*α* = 0.71). These behaviors involved positioning the stepfather as a substitute for the biological father and preventing interaction with the biological father. Factor 3, Imposing Rules and Values, consisted of four items with factor loadings ranging from 0.62 – 0.72 (*α* = 0.83). These behaviors included imposing values and rules and being strict with children.

#### 3.1.3 Nonresidential Fathers' Parenting Behaviors

EFA was conducted using an initial set of 10 items, and a two‐factor model emerged. The loadings of the items are shown in Table S4. Factor 1, Displaying Warmth, consisted of five items with factor loadings ranging from 0.84 – 0.94 (*α* = 0.95). These behaviors included listening carefully, fulfilling wishes, and expressing enthusiasm for visitation. Factor 2, Imposing Rules and Values, consisted of five items with factor loadings ranging from 0.66 – 0.93 (*α* = 0.92). These indicators included imposing values and rules and being strict with children.

#### 3.1.4 Association Between Stepparenting and Parenting Behaviors and PBI

Following EFA, partial correlations were used to assess the relationship between each emergent factor and subscales from the well‐established PBI, while controlling for the other subscales within the same Stepparenting and Parenting Behaviors dimension (see Table S5). Expectedly, the PBI subscale of Care was strongly correlated with the emergent factor of stepfathers' Engaging in Affinity‐Seeking and Advocating for Stepchild (*r* = 0.84, *p* < 0.01), residential mothers' Maintaining Close Parent–Child Bonds and Supporting Stepparent–Child Relationship (*r* = 0.67, *p* < 0.01), and nonresidential fathers' Displaying Warmth (*r* = 0.84, *p* < 0.01). Similarly, the PBI subscale of Denial of Psychological Autonomy was moderately correlated with the emergent factor of Imposing Rules and Values (stepfather: *r* = 0.43, *p* < 0.01; residential mother: *r* = 0.33, *p* < 0.01; nonresidential father: *r* = 0.64, *p* < 0.01). In contrast, the PBI subscale of Encouragement of Behavioral Freedom showed little to no correlation with the emergent factor of Replacing the Nonresidential Biological Father (stepfather: *r* = −0.17, *p* < 0.01; residential mother: *r* = −0.05, *n.s*.). In all cases, the partial correlations were controlled for the other subscales within the same parental domain.

### 3.2 The Latent Profiles

#### 3.2.1 Model Comparisons

We used 12 subscales and estimated solutions with one to six profiles. Table S6 shows model fit statistics for determining the optimal number of profiles. AIC, BIC, and aBIC values were lowest for the six‐profile solution. Entropy was highest for the six‐profile solution, followed by the five‐ and four‐profile solutions. However, VLRT and LMR tests were not significant for the five‐profile solution; therefore, the four‐profile solution provided a significantly better fit to the data.

#### 3.2.2 Patterns of Stepparenting, Parenting Behaviors, and the Dynamics of Stepcouple and Co‐Parenting Relationships

**FIGURE 1:**
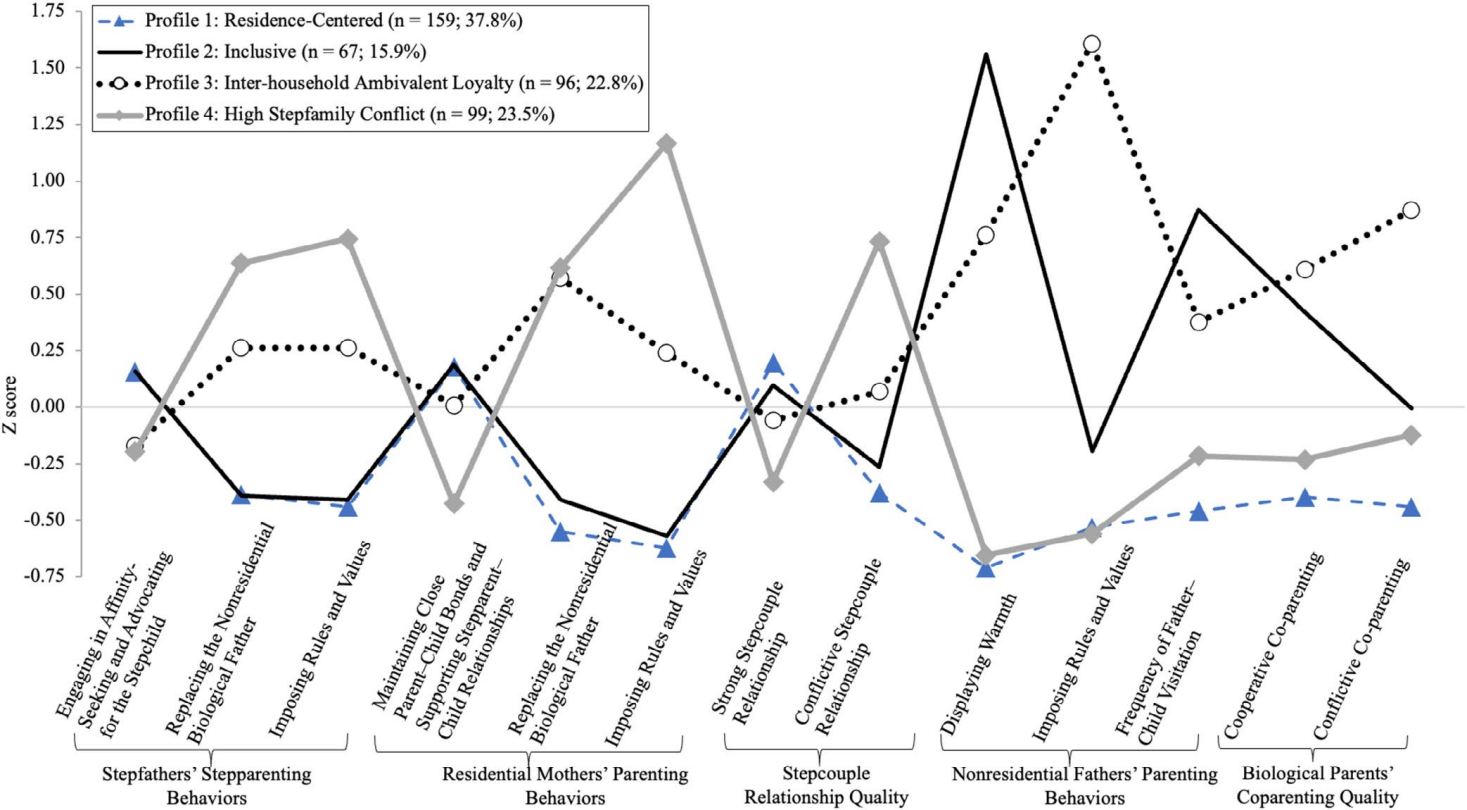
Patterns of stepparenting, parenting behaviors, and the dynamics of stepcouple and co‐parenting relationships.

Figure 1 shows differences among profiles using Z‐scores for each variable. In Profile 1, Residence‐Centered pattern (*n* = 159; 37.8%), EAs reported below‐average scores for residential mothers' and stepfathers' Replacing Nonresidential Biological Father (*Z* = −0.38, −0.55, respectively), Imposing Rules and Values (*Z* = −0.44, −0.62, respectively), and nonresidential fathers' parenting behaviors (Z = −0.71 to −0.46). In Profile 2, Inclusive pattern (*n* = 67; 15.9%), EAs reported similar scores to those in Profile 1 for stepfathers' stepparenting and residential mothers' parenting behaviors. However, they reported above‐average scores for nonresidential fathers' Displaying Warmth (*Z* = 1.56), Frequency of Father–Child Visitation (*Z* = 0.87), and biological parents' Cooperative Co‐parenting (*Z* = 0.42). In Profile 3, Inter‐household Ambivalent Loyalty (*n* = 96; 22.8%), EAs reported above‐average scores for nonresident fathers' Displaying Warmth and Imposing Rules and Values (*Z* = 0.76, 1.61, respectively) and biological parents' Conflictive Co‐parenting (*Z* = 0.87). In Profile 4, High Stepfamily Conflict pattern (*n* = 99; 23.5%), EAs reported above‐average scores for mothers' and stepfathers' Replacing Nonresidential Biological Father (*Z* = 0.64, 0.62, respectively), Imposing Rules and Values (*Z* = 0.74, 1.17, respectively), and Conflictive Stepcouple relationship (*Z* = 0.73). In contrast, they reported below‐average scores for nonresidential fathers' parenting behaviors (*Z* = −0.66 to −0.22).

### 3.3 Predictors of Profile Membership

Tables S7 and S8 summarize the covariates, including counts and means, for each profile. Table 1 shows covariates associated with profile membership. Male EAs were less likely than female EAs to be in Profile 3 (Inter‐household Ambivalent Loyalty) or Profile 4 (High Stepfamily Conflict) than in Profile 2 (Inclusive; OR = 0.21; OR = 0.36), and less likely to be in Profile 3 than in Profile 1 (Residence‐Centered; OR = 0.34). EAs who experienced parental divorce at a younger age were less likely to be in Profile 1 and Profile 4 than in Profile 2 (OR = 0.82; OR = 0.88) and were more likely to be in Profile 3 than in Profile 1 (OR = 1.16). EAs who had at least one full sibling were less likely to be in Profile 1, Profile 3, or Profile 4 than in Profile 2 (OR = 0.42; OR = 0.34; OR = 0.41). EAs who had at least one half‐sibling were less likely to be in Profile 3 than in Profile 2 (OR = 0.43). EAs who experienced the breaking up of their biological mother and stepfather were more likely to be in Profile 3 or Profile 4 than in Profile 2 (OR = 3.54; OR = 2.40) and more likely to be in Profile 3 or Profile 4 than in Profile 1 (OR = 3.95; OR = 2.68).

**TABLE 1:** Predictors of profile membership.

Ref.	Profile 2: Inclusive									Profile 1: Residence‐centered						Profile 3: Inter‐household Ambivalent Loyalty		
	Profile 1: Residence‐Centered			Profile 3: Inter‐household Ambivalent Loyalty			Profile 4: High Stepfamily Conflict			Profile 3: Inter‐household Ambivalent Loyalty			Profile 4: High Stepfamily Conflict			Profile 4: High Stepfamily Conflict		
	OR	95% CI		OR	95% CI		OR	95% CI		OR	95% CI		OR	95% CI		OR	95% CI	
EAs' sex (male: 0, female: 1)	0.62	0.26	1.52	**0.21**	0.08	0.52	**0.36**	0.14	0.89	**0.34**	0.18	0.64	0.57	0.30	1.11	1.70	0.89	3.25
EAs' age at biological parents' divorce	**0.82**	0.73	0.93	0.96	0.84	1.09	**0.88**	0.77	0.99	**1.16**	1.04	1.29	1.06	0.96	1.17	0.92	0.83	1.01
EAs' age when entering stepfamily	1.08	0.96	1.21	1.03	0.90	1.17	0.99	0.88	1.12	0.95	0.86	1.05	0.92	0.84	1.01	0.97	0.88	1.07
Any full siblings	**0.42**	0.19	0.90	**0.34**	0.14	0.80	**0.41**	0.18	0.95	0.80	0.43	1.50	0.99	0.55	1.81	1.24	0.66	2.33
Any stepsiblings	0.76	0.19	3.07	1.11	0.25	4.98	0.81	0.19	3.46	1.46	0.43	4.94	1.07	0.30	3.87	0.73	0.22	2.40
Any half‐siblings	0.81	0.43	1.52	**0.43**	0.19	0.94	0.81	0.40	1.62	0.53	0.28	1.02	1.00	0.56	1.82	1.90	0.95	3.79
Stepcouple breaking up	0.90	0.41	1.98	**3.54**	1.61	7.76	**2.40**	1.10	5.24	**3.95**	2.11	7.39	**2.68**	1.40	5.13	0.68	0.36	1.27
Biological fathers' remarriage	0.88	0.46	1.68	1.55	0.75	3.23	1.47	0.73	2.97	1.77	0.98	3.21	1.68	0.94	3.01	0.95	0.51	1.77

*Note:* Bold indicates statistically significant results (95% CI does not include 1).

Abbreviations: CI, confidence interval; OR, odds ratio.

### 3.4 Effects of Profile Membership on EAs' Outcomes

Table 2 presents means and standard errors for each profile regarding EAs' outcomes. Self‐esteem was significantly higher in Profile 2 (Inclusive) than in Profile 4 (High Stepfamily Conflict). Levels of depression and anxiety were significantly higher in Profile 3 (Inter‐household Ambivalent Loyalty) and Profile 4 than in Profile 1 (Residence‐Centered) and Profile 2. Proactive aggression was significantly higher in Profile 3 than in any other profile, and in Profile 4 than in Profile 2. Reactive aggression was significantly higher in Profile 3 and Profile 4 than in Profile 1 and Profile 2.

**TABLE 2:** Profile‐specific means and standard errors of EAs' outcomes.

	Profile 1: Residence‐Centered		Profile 2: Inclusive		Profile 3: Inter‐household Ambivalent Loyalty		Profile 4: High Stepfamily Conflict		Significant profile differences (*p* < 0.05)
	M	SE	M	SE	M	SE	M	SE	
Self‐esteem	2.85	(0.07)	2.98	(0.11)	2.87	(0.06)	2.71	(0.07)	2 > 4
Depression and anxiety	11.43	(0.99)	9.44	(1.27)	17.63	(0.99)	18.00	(1.30)	3, 4 > 1, 2
Proactive aggression	2.31	(0.06)	2.19	(0.10)	2.82	(0.07)	2.53	(0.10)	3 > 1, 2, 4; 4 > 2
Reactive aggression	2.72	(0.08)	2.55	(0.13)	2.96	(0.07)	3.22	(0.13)	3, 4 > 1, 2

*Note:* Means and group differences were estimated using the 3‐step procedure.

## 4 Discussion

This study aimed to identify patterns in stepparenting, parenting behaviors, and the quality of stepcouple and biological parents' co‐parenting relationships, and their associations with sociodemographic factors and psychological outcomes among Japanese emerging adults (EAs). Four distinct profiles were identified, with the Residence‐Centered pattern (37.8%) being the most common and the Inclusive pattern (15.9%) the least prevalent. The Residence‐Centered and Inclusive patterns were associated with higher self‐esteem, lower depression and anxiety, and lower aggression compared to the Inter‐household Ambivalent Loyalty and High Stepfamily Conflict patterns. Although no significant difference existed between the Residence‐Centered and Inclusive patterns, the Inclusive pattern was associated with higher self‐esteem and lower proactive aggression compared to the High Stepfamily Conflict pattern.

The Residence‐Centered pattern was characterized by high‐quality relationships with the co‐residential parent, whereas the nonresidential father was largely uninvolved, suggesting that he could be excluded from the family system. It may reflect a closed household boundary which is associated with limited involvement of the nonresidential parent. Notably, this configuration aligns closely with Nozawa's (2015a, 2020) “scrap and build” household model, in which the prior family structure is dismantled and replaced by a new unit consisting of the custodial parent, child, and stepparent. The prevalence of this pattern—accounting for 37.8% of the sample—suggests that it remains the dominant stepfamily form in Japan. This may be influenced by institutional factors, such as the sole custody system and stepparent adoption policies, which tend to reinforce exclusive bonds within the new household while limiting the role of the noncustodial parent. However, it is noteworthy that, under this pattern, EAs showed relatively good psychological adjustment. The pattern may include cases where the biological parents divorced at an early stage and the residential biological mother and stepfather gradually engaged in affinity‐seeking behaviors without forcing the stepparent to replace the biological father. Prior research on stepparent–child relationships has identified similar patterns, such as “continuously accepting as a parent” (Nozawa 2015a) and “accepting as a parent” (Ganong et al. 2011). The current pattern may fall into one of these categories and be an example of what works for stepfamilies.

The Inclusive pattern involved sustained and positive relationships with the nonresidential father, indicating that he was included within the family boundary and integrated into the family system. Predictors of the Inclusive pattern included being female, having full siblings, and being older at the time of parental divorce, suggesting that individuals in this profile may benefit from sibling support and positive interactions with their three parental figures. Compared to Western studies, where 26%–55% of individuals in similar profiles maintain strong relationships with all parental figures (Amato et al. 2016; Egginton et al. 2021; Jensen 2017), the proportion of individuals in the Inclusive pattern in this study was lower (15.9%). This discrepancy may be related to Japan's sole custody system and limited frequency of visitation exchanges.

The Inter‐household Ambivalent Loyalty and High Stepfamily Conflict patterns were associated with poorer psychological outcomes. Particularly, the Inter‐household Ambivalent Loyalty pattern showed significantly higher proactive aggression than the other profiles. This pattern was characterized by the absence of half‐siblings and a high rate of stepcouple breakup. The relatively high level of warmth displayed by the nonresidential biological father may result from the stepcouple's breakup and the child's increased dependence on the biological father. However, EAs in this profile also perceived their biological father as imposing rules and values, leading to a conflictual relationship with both biological parents. Additionally, nonresidential fathers appeared more likely to have remarried in this pattern than in other patterns, which may result in their being less available to the EA either emotionally or practically. It is also possible that nonresidential fathers maintain contact out of obligation, but hesitate to intervene in the maternal‐stepfather household, which is characterized by a relationship that is superficial and lacks substantive support. Furthermore, the high level of biological parent's conflictive co‐parenting may be associated with greater loyalty conflicts. Consequently, the combination of conflict between the stepcouples and biological parents, and the instability of the EAs relationships with their biological fathers may all contribute to poorer psychological adjustment.

The High Stepfamily Conflict pattern was associated with a distant relationship with the biological father and high levels of stepcouple conflict. Furthermore, EAs perceived the biological mother as providing limited emotional support while imposing strict rules and values. Given the critical role of the residential biological parent (Cartwright and Seymour 2002; Nozawa 2015b; Weaver and Coleman 2010), a weak relationship with the biological mother in this profile may be associated with poorer psychological outcomes.

Additionally, the four profiles identified in this study closely align with relationship quality categorizations found in Western studies (Ganong and Sanner 2023)—the Residence‐Centered pattern corresponds to Substitution, the Inclusive pattern to Close to Both or All, the Inter‐household Ambivalent Loyalty pattern to Retaining, and the High Stepfamily Conflict pattern to Close to Neither or None. The Retaining pattern—characterized by a strong relationship only with the separated biological father—was associated with poorer psychological outcomes than other patterns (Egginton et al. 2021; Jensen et al. 2017). Although the exact cause remained unclear, loyalty conflicts were suggested as a possible explanation. By assessing both the positive and negative dimensions of behavioral and relational quality, this study was able to identify patterns that had not been observed in prior research.

### 4.1 Limitations and Research Implications

This study has several limitations. First, its cross‐sectional and retrospective design may introduce recall bias (Scott and Alwin 1998). Given the positive nature of the current parent–child relationship, EAs may have retrospectively viewed their past relationship in a similarly favorable light. Future research should adopt a longitudinal approach to address this issue. Second, the sample was not fully representative. Most participants were women, and the study focused only on EAs who lived with their biological mother and stepfather. Future research should incorporate more diverse stepfamily structures to improve generalizability. Third, this study may not fully capture the effects of recent Japanese family law reforms. For instance, visitation rights were legally established in 2011, but most participants had already entered stepfamilies before this change. It is crucial to conduct further studies to assess the impact of forthcoming legal reforms—such as the introduction of joint legal custody in 2026.

### 4.2 Clinical Implications

This study provides important clinical insights by applying a family systems perspective to stepfamily relationships and EAs psychological adjustment. Based on the family systems theory, which views the family as an interconnected system, this study assessed how dynamics among residential parents, stepparents, and nonresidential parents are linked to adjustment. Its findings showed that these interrelated dynamics can both support and complicate EAs' adjustment. For example, maintaining a positive relationship with a nonresidential father may support psychological well‐being, but it may also trigger loyalty conflicts with the residential parent if the child's best interest is not a shared goal between the nonresidential and residential parents. These results highlight the importance of addressing both dyadic relationships and the broader family system in clinical practice.

Its findings also indicated clear associations between specific relationship patterns and psychological adjustment. In particular, the Inter‐household Ambivalent Loyalty and High Stepfamily Conflict patterns were linked to lower self‐esteem and higher aggression among EAs, suggesting that these groups may require prioritized clinical attention. Moreover, the differences between profiles were clearly marked by negative relational features (i.e., Replacing the Nonresidential Biological Parent with the Stepparent; Imposing Rules and Values), as well as positive ones (i.e., Engaging Affinity‐Seeking). Although these negative relational characteristics may not independently act as risk factors, they were more frequently observed in profiles associated with poorer psychological outcomes. These findings provide suggestions as to what can be particularly effective for stepparents. Important implications for future psychoeducational and clinical interventions could be that the stepparent should try to be a friendly affinity‐seeker, rather than a rule‐imposing parental figure, who replaces the biological parent. This behavioral pathway might help prevent stepchildren's long‐term maladjustment.

Finally, Japan has a limited number of clinicians who are familiar with what works and what does not work in stepfamilies (Ganong et al. 2025; Papernow 2013). Therefore, clinical training programs should incorporate such knowledge to better prepare practitioners for working with these family systems. Japanese clinicians also tend to understand stepfamilies through a rigid framework, often viewing them as a “scrap‐and‐build” household model. As Papernow (2013) emphasized, effective clinical interventions for stepfamilies require paying attention not only to the residential household but also to nonresidential parents. Expanding clinicians' perspectives to include the entire stepfamily system, by adding both the former and present partners across households, is essential for providing support that aligns with the unique relational challenges in these families.

## Disclosure

We employed ChatGPT (GPT‐ 4o, OpenAI, 2025) to enhance the language and ensure that the paper conforms to the standards of scholarly journals. Please note that ChatGPT was not used to generate individual sentences or ideas in this study.

## Conflicts of Interest

The authors declare no conflicts of interest.

## Supporting information


**Tables S1–S8:** famp70071‐sup‐0001‐TablesS1‐S8.docx.

## Data Availability

The data that support the findings of this study are available from the corresponding author upon reasonable request.
