# Supplementary material for: Stepfamily Dynamics and Emerging Adults' Adjustment in Japan: Four Patterns Affecting Stepchildren's Outcomes
Source: Fam Process. 2025 Sep 26;64(4):e70071. doi: 10.1111/famp.70071 (PMC12464621; doi:10.1111/famp.70071)
Supplement: Supplementary file 1 — Tables S1–S8: famp70071‐sup‐0001‐TablesS1‐S8.docx. [file FAMP-64-0-s001.docx]

**Supplemental Materials**

**Table S1**

*Effective and Ineffective Stepparenting and Parenting Behaviors*

|  | Stepparent | Biological parent | |
| --- | --- | --- | --- |
|  |  | Resident | Nonresident |
| Effective | Engaging in Affinity-Seeking | Maintaining Close Parent–Child Bonds (Displaying Warmth, Protecting One-on-One Time) | |
|  | Advocating for the Stepchild | Supporting Stepparent–Child Relationship Development |  |
| Ineffective | Replacing the Nonresidential Biological Parent with the Stepparent | |  |
|  | Imposing Rules and Values | | |

**Table S2**

*Results From an Exploratory Factor Analysis of Stepfathers*’ *Stepparenting Behaviors Subscale*

|  |  | Factor loading | | |  |  |
| --- | --- | --- | --- | --- | --- | --- |
|  |  | 1 | 2 | 3 | *M* | *SD* |
| Factor 1: Engaging in Affinity-Seeking and Advocating for the Stepchild | | | | |  |  |
|  | My stepfather had one-on-one conversations with me. （私と一対一で話をしてくれた） | **.86** | -.08 | .11 | 2.52 | 1.07 |
|  | My stepfather listened to me attentively. （私の話をよく聴いてくれた） | **.85** | -.09 | -.03 | 2.45 | 1.09 |
|  | My stepfather tried to understand me. （私のことを理解してくれようとした） | **.82** | -.14 | -.02 | 2.51 | 1.08 |
|  | My stepfather played with me one-on-one. （私と一対一で遊んでくれた） | **.81** | -.15 | .13 | 2.45 | 1.09 |
|  | My stepfather granted my wishes (e.g., playing, traveling, gifts). （私が希望したこと（遊び、旅行、プレゼント等）を叶えてくれた） | **.79** | -.13 | .06 | 2.58 | 1.07 |
|  | My stepfather explained my mother's thoughts to help me understand her perspective. （母親が考えていることを私が分かるよう話してくれた） | **.72** | .22 | -.08 | 2.15 | 1.05 |
|  | My stepfather said what I wanted to say to my mother. （私が母親に言いたいことを代弁してくれた） | **.71** | .28 | -.10 | 2.06 | 1.06 |
|  | My stepfather mediated when my mother and I disagreed. （私と母親との意見があわなかった時に間に入ってくれた） | **.71** | .26 | -.06 | 2.08 | 1.01 |
| Factor 2: Replacing the Nonresidential Biological Father | | | | |  |  |
|  | My stepfather prevented me from having contact with my biological father. （私の実の父親との交流を妨げた） | -.04 | **.79** | -.06 | 1.69 | 0.94 |
|  | My stepfather became moody or angry whenever my biological father was mentioned. （私の実の父親の話題になると不機嫌になったり、怒ったりした） | -.04 | **.72** | .01 | 1.74 | 0.97 |
|  | My stepfather told me I didn’t have to see my biological father. （私の実の父親とは会わなくてよいと言った） | .08 | **.68** | -.01 | 1.81 | 1.03 |
|  | My stepfather insisted that he was my father. （自分のことを父親である、と言いきかせた） | .05 | **.67** | .13 | 1.76 | 0.95 |
|  | My stepfather asked me to call him "Dad." （自分のことをお父さん（パパ）と呼ぶよう求めた） | -.02 | **.60** | .15 | 1.79 | 1.04 |
| Factor 3: Imposing Rules and Values | | | | |  |  |
|  | My stepfather tried to discipline me strictly. （私を厳しくしつけようとした） | .07 | -.02 | **.85** | 2.19 | 1.14 |
|  | My stepfather made rules and expected me to follow them. （決まり事を作って私にその決まり事を守らせようとした） | .11 | .01 | **.80** | 2.17 | 1.11 |
|  | My stepfather was harsh with me. （私に厳しくあたってきた） | -.06 | .08 | **.76** | 2.20 | 1.12 |
|  | My stepfather tried to impose his values on me. （私に価値観を押し付けてきた） | -.17 | .22 | **.58** | 2.12 | 1.14 |
|  | Interfactor correlation: Factor 2 | .07 | .58 |  |  |  |
|  | Factor 3 | -.24 |  |  |  |  |

*Note.* *N* = 421. The factor extraction method is the maximum likelihood method with Promax rotation. Factor loadings above .40 are in bold. Items in parentheses are written in Japanese for reference. **Table S3**

*Results From an Exploratory Factor Analysis of Residential Mothers’ Parenting Behaviors Subscale*

|  | Item | Factor loading | | |  |  |
| --- | --- | --- | --- | --- | --- | --- |
|  |  | 1 | 2 | 3 | *M* | *SD* |
| Factor 1: Maintaining Close Parent–Child Bonds and Supporting Stepparent–Child Relationships | | | | | |  |
|  | If my stepfather's involvement was inappropriate, my mother helped me. （継父の関わりが不適切であれば私のことを助けてくれた） | **.83** | -.14 | .21 | 2.52 | 1.05 |
|  | My mother stepped in when my stepfather and I disagreed. （私と継父との意見があわなかった時に間に入ってくれた） | **.78** | -.09 | .13 | 2.43 | 1.03 |
|  | My mother protected me from my stepfather. （継父から私のことを守ってくれた） | **.77** | -.14 | .16 | 2.49 | 1.01 |
|  | My mother spoke on my behalf to my stepfather. （私が継父に言いたいことを代弁してくれた） | **.75** | .07 | .02 | 2.46 | 1.08 |
|  | My mother thought of me first. （私のことを第一に考えてくれていた） | **.74** | -.09 | -.14 | 2.72 | 1.07 |
|  | My mother took the time to watch over my relationship with my stepfather. （私と継父との関係を時間をかけて見守ってくれた） | **.73** | .07 | -.09 | 2.52 | 1.02 |
|  | My mother was considerate and asked me about my thoughts and feelings regarding my relationship with my stepfather and my new family. （継父との関係や新しい家族に関する私の考えや気持ちについて気を配り、関心をもってたずねてくれた） | **.71** | .18 | -.11 | 2.36 | 1.05 |
|  | When I was having trouble with my stepfather, my mother explained his thoughts to help me understand. （私が継父とうまくいかないときに、継父が考えていることを私が理解できるように伝えてくれた） | **.70** | .18 | -.02 | 2.31 | 1.04 |
|  | I felt that my mother made time with me a top priority. （私との時間を最優先にしてくれたと感じた） | **.70** | .07 | -.15 | 2.48 | 1.06 |
| Factor 2: Replacing the Nonresidential Biological Father | | | |  |  |  |
|  | My mother became moody or angry when the topic of my biological father came up. （実の父親の話題になると不機嫌になったり、怒ったりした） | -.07 | **.63** | .05 | 1.90 | 1.05 |
|  | My mother insisted that my stepfather was my father. （継父のことを私の父親である、と言いきかせた） | .07 | **.62** | .05 | 1.85 | 0.98 |
|  | I felt that my mother thought of my stepfather first. （継父のことを第一に考えているような気がした） | -.02 | **.61** | .01 | 1.96 | 0.99 |
|  | My mother said I didn't have to see my biological father. （実の父親とは会わなくてよいと言った） | .12 | **.46** | -.03 | 2.33 | 1.16 |
|  | My mother asked me to call my stepfather "Dad." （継父のことをお父さん（パパ）と呼ぶよう求めた） | .04 | **.41** | .20 | 1.99 | 1.11 |
| Factor 3: Imposing Rules and Values | | | |  |  |  |
|  | My mother made rules and expected me to follow them. （決まり事を作って私にその決まり事を守らせようとした） | .15 | .02 | **.72** | 2.19 | 1.07 |
|  | My mother was strict with me. （私に厳しくあたってきた） | -.11 | .12 | **.70** | 2.00 | 1.02 |
|  | My mother tried to discipline me strictly. （私を厳しくしつけようとした） | .04 | -.01 | **.70** | 2.13 | 1.07 |
|  | My mother tried to impose her values on me. （私に価値観を押し付けてきた） | -.12 | .21 | **.62** | 2.02 | 1.10 |
|  | Interfactor correlation: Factor 2 | .00 | .65 |  |  |  |
|  | Factor 3 | -.17 |  |  |  |  |

*Note.* *N* = 421. The factor extraction method is the maximum likelihood method with Promax rotation. Factor loadings above .40 are in bold. Items in parentheses are written in Japanese for reference.

**Table S4**

*Results From an Exploratory Factor Analysis of Nonresidential Fathers’ Parenting Behaviors*

*Subscale*

|  |  | Factor loading | |  |  |
| --- | --- | --- | --- | --- | --- |
|  |  | 1 | 2 | *M* | *SD* |
| Factor 1: Displaying Warmth | | | | |  |
|  | My father listened to me. （私の話をよく聴いてくれた） | **.94** | -.05 | 1.77 | 1.08 |
|  | My father tried to understand me. （私のことを理解してくれようとした） | **.90** | .01 | 1.72 | 1.06 |
|  | My father granted my wishes (e.g., playing, traveling, gifts). （私が希望したこと（遊び、旅行、プレゼント等）を叶えてくれた） | **.87** | -.02 | 1.82 | 1.15 |
|  | My father had one-on-one conversations with me. （私と一対一で話をしてくれた） | **.86** | .06 | 1.84 | 1.15 |
|  | My father looked forward to seeing and talking to me. （私と会ったり話をすることを楽しみにしてくれていた） | **.84** | .04 | 1.86 | 1.18 |
| Factor 2: Imposing Rules and Values | | | | |  |
|  | My father was strict with me. （私に厳しくあたってきた） | -.09 | **.93** | 1.57 | 0.98 |
|  | My father imposed his values on me. （私に価値観を押し付けてきた） | -.01 | **.87** | 1.54 | 0.91 |
|  | My father tried to discipline me strictly. （私を厳しくしつけようとした） | .01 | **.85** | 1.52 | 0.95 |
|  | My father made rules and expected me to follow them. （決まり事を作って私にその決まり事を守らせようとした） | .05 | **.83** | 1.54 | 0.95 |
|  | My father wasn't interested in seeing me or talking to me. （私と会ったり話をすることに関心がなかった） | .10 | **.66** | 1.56 | 0.94 |
|  | Interfactor correlation: Factor 2 | .50 |  |  |  |

*Note.* *N* = 421. The factor extraction method is the maximum likelihood method with Promax rotation. Factor loadings above .40 are in bold. Items in parentheses are written in Japanese for reference.

**Table S5**

*Association between Stepparenting and Parenting Behaviors and PBI*

|  |  |  | Parental Bonding Instrument (PBI) | | | | | | | | | | |
| --- | --- | --- | --- | --- | --- | --- | --- | --- | --- | --- | --- | --- | --- |
|  |  |  | Care ^a^ | | |  | Denial of Psychological Autonomy ^b^ | | |  | Encouragement of Behavioral Freedom ^c^ | | |
| Stepfathers’ Stepparenting Behaviors | |  |  |  |  |  |  |  |  |  |  |  |  |
|  | Engaging in Affinity-Seeking and Advocating for the Stepchild ^d^ |  | *r* = _ad.ef_ | .84 | ** |  | *r* = _bd.ef_ | −.13 | ** |  | *r* = _cd.ef_ | .70 | ** |
|  | Replacing the Nonresidential Biological Father ^e^ |  | *r* = _ae.df_ | −.16 | ** |  | *r* = _be.df_ | .21 | ** |  | *r* = _ce.df_ | −.17 | ** |
|  | Imposing Rules and Values ^f^ |  | *r* = _af.de_ | −.22 | ** |  | *r* = _bf.de_ | .43 | ** |  | *r* = _cf.de_ | −.30 | ** |
| Residential Mothers’ Parenting Behaviors | |  |  |  |  |  |  |  |  |  |  |  |  |
|  | Maintaining Close Parent–Child Bonds and  Supporting Stepparent–Child Relationships ^g^ |  | *r* = _ag.hi_ | .67 | ** |  | *r* = _bg.hi_ | −.14 | ** |  | *r* = _cg.hi_ | .55 | ** |
|  | Replacing the Nonresidential Biological Father ^h^ |  | *r* = _ah.gi_ | −.07 |  |  | *r* = _bh.gi_ | .07 |  |  | *r* = _ch.gi_ | −.05 |  |
|  | Imposing Rules and Values ^i^ |  | *r* = _ai.gh_ | −.28 | ** |  | *r* = _bi.gh_ | .33 | ** |  | *r* = _ci.gh_ | −.33 | ** |
| Nonresidential Fathers’ Parenting Behaviors | |  |  |  |  |  |  |  |  |  |  |  |  |
|  | Displaying Warmth ^j^ |  | *r* = _aj.k_ | .84 | ** |  | *r* = _bj.k_ | .26 | ** |  | *r* = _cj.k_ | .80 | ** |
|  | Imposing Rules and Values ^k^ |  | *r* = _ak.j_ | −.21 | ** |  | *r* = _bk.j_ | .64 | ** |  | *r* = _ck.j_ | .11 | * |

** *p* <.01. * *p* <.05.

**Table S6**

*Model Fit Statistics for Determining Number of Profiles*

| Profile | AIC | BIC | aBIC | VLRT | *p* | LMR | *p* | BLRT | *p* | Entropy | Smallest Profile Size (%) |
| --- | --- | --- | --- | --- | --- | --- | --- | --- | --- | --- | --- |
| 1 | 15570.69 | 15675.80 | 15593.29 |  |  |  |  |  |  |  |  |
| 2 | 14944.67 | 15106.37 | 14979.44 | −7759.34 | .000 | 646.38 | .000 | −7759.34 | .000 | .95 | 162 (38.48%) |
| 3 | 14569.51 | 14787.81 | 14616.45 | −7432.33 | .024 | 398.45 | .025 | −7432.33 | .000 | .96 | 62 (14.73%) |
| 4 | 14205.31 | 14480.21 | 14264.43 | −7230.75 | .000 | 387.61 | .000 | −7230.75 | .000 | .93 | 67 (15.91%) |
| 5 | 14045.93 | 14377.43 | 14117.22 | −7034.66 | .121 | 185.19 | .126 | −7034.66 | .000 | .93 | 51 (12.11%) |
| 6 | 13912.98 | 14301.07 | 13996.43 | −6940.97 | .561 | 159.08 | .564 | −6940.97 | .000 | .94 | 16 (3.80%) |

*Note.* AIC = Akaike’s information criterion; BIC = Bayesian information criterion; aBIC = adjusted BIC; VLRT = Vuong-Lo-Mendell-Rubin likelihood ratio test; LMR = Lo-Mendell-Rubin likelihood ratio test; BLRT = Bootstrap Likelihood Ratio Test.

**Table S7**

*Descriptive Statistics of Four Profiles and Covariates (Including χ² Tests)*

|  |  | Profile 1: Residence-Centered | Profile 2: Inclusive | Profile 3:  Inter-household Ambivalent Loyalty | Profile 4:  High Stepfamily Conflict | *n* | χ2 |
| --- | --- | --- | --- | --- | --- | --- | --- |
| EAs’ sex | male | **29** | **10** | **40** | 27 | 106 | 21.89 |
|  |  | **-2.6** | **-2.1** | **4.2** | 0.5 |  | *ｐ* < .01 |
|  | female | **130** | **57** | **56** | 72 | 315 |  |
|  |  | **2.6** | **2.1** | **-4.2** | -0.5 |  |  |
| Any full Siblings | No | 66 | **13** | 35 | 38 | 152 | 10.34 |
|  |  | 1.8 | **-3.1** | 0.1 | 0.5 |  | *p* < .05 |
|  | Yes | 93 | **54** | 61 | 61 | 269 |  |
|  |  | -1.8 | **3.1** | -0.1 | -0.5 |  |  |
| Any stepsiblings | No | 150 | 63 | 88 | 93 | 394 | 0.78 |
|  |  | 0.5 | 0.2 | -0.9 | 0.2 |  | *n.s.* |
|  | Yes | 9 | 4 | 8 | 6 | 27 |  |
|  |  | -0.5 | -0.2 | 0.9 | -0.2 |  |  |
| Any half-siblings | No | 91 | 40 | **72** | 57 | 260 | 9.36 |
|  |  | -1.5 | -0.4 | **3.0** | -1.0 |  | *ｐ* < .05 |
|  | Yes | 68 | 27 | **24** | 42 | 161 |  |
|  |  | 1.5 | 0.4 | **-3.0** | 1.0 |  |  |
| Stepcouple breaking up | No | **130** | 53 | **52** | **62** | 297 | 27.37 |
|  |  | **3.9** | 1.7 | **-4.0** | **-2.0** |  | *ｐ* < .01 |
|  | Yes | **29** | 14 | **44** | **37** | 124 |  |
|  |  | **-3.9** | -1.7 | **4.0** | **2.0** |  |  |
| Biological fathers’ remarriage | No | **105** | 41 | **46** | 53 | 245 | 9.32 |
|  |  | **2.5** | 0.5 | **-2.3** | -1.1 |  | *ｐ* < .01 |
|  | Yes | **54** | 26 | **50** | 46 | 176 |  |
|  |  | **-2.5** | -0.5 | **2.3** | 1.1 |  |  |

*Note*. Values in the upper row represent sample sizes (*n*), and values in the lower row represent adjusted standardized residuals. Significant residuals (*p* < .05, |z| > 1.96) are shown in bold. **Table S8**

*Descriptive Statistics of Four Profiles and Covariates (Including ANOVA)*

|  | Full sample | Profile 1: Residence-Centered | Profile 2: Inclusive | Profile 3:  Inter-household Ambivalent Loyalty | Profile 4:  High Stepfamily Conflict | *F* |  |
| --- | --- | --- | --- | --- | --- | --- | --- |
| EAs’ age at biological parents’ divorce | 4.91 | 4.12 | 6.13 | 5.98 | 4.31 | 9.20 | 2, 3 > 1, 4 |
|  | (3.64) | (3.32) | (3.62) | (4.04) | (3.28) | *ｐ* < .01 |  |
| EAs’ age when entering stepfamily | 8.92 | 8.86 | 9.39 | 9.42 | 8.21 | 2.23 |  |
|  | (3.65) | (3.63) | (3.62) | (3.85) | (3.44) | *p* = .084 |  |

*Note*. The upper row represents means, and the lower row represents standard deviations. Different superscripts indicate significant group differences (*p* < .05, Tukey’s HSD test).
